# Understanding Factors That Modulate the Establishment of HIV Latency in Resting CD4+ T-Cells *In Vitro*

**DOI:** 10.1371/journal.pone.0158778

**Published:** 2016-07-06

**Authors:** Jenny L. Anderson, Talia M. Mota, Vanessa A. Evans, Nitasha Kumar, Simin D. Rezaei, Karey Cheong, Ajantha Solomon, Fiona Wightman, Paul U. Cameron, Sharon R. Lewin

**Affiliations:** 1 Peter Doherty Institute for Infection and Immunity, The University of Melbourne, Melbourne, Victoria, Australia; 2 Department of Infectious Diseases, Monash University and Alfred Hospital, Melbourne, Victoria, Australia; Jackson Laboratory, UNITED STATES

## Abstract

Developing robust *in vitro* models of HIV latency is needed to better understand how latency is established, maintained and reversed. In this study, we examined the effects of donor variability, HIV titre and co-receptor usage on establishing HIV latency *in vitro* using two models of HIV latency. Using the CCL19 model of HIV latency, we found that in up to 50% of donors, CCL19 enhanced latent infection of resting CD4+ T-cells by CXCR4-tropic HIV in the presence of low dose IL-2. Increasing the infectious titre of CXCR4-tropic HIV increased both productive and latent infection of resting CD4+ T-cells. In a different model where myeloid dendritic cells (mDC) were co-cultured with resting CD4+ T-cells, we observed a higher frequency of latently infected cells *in vitro* than CCL19-treated or unstimulated CD4+ T-cells in the presence of low dose IL-2. In the DC-T-cell model, latency was established with both CCR5- and CXCR4-tropic virus but higher titres of CCR5-tropic virus was required in most donors. The establishment of latency *in vitro* through direct infection of resting CD4+ T-cells is significantly enhanced by CCL19 and mDC, but the efficiency is dependent on virus titre, co-receptor usage and there is significant donor variability.

## Introduction

Long-lived, latently infected memory CD4+ T-cells persist in people living with HIV on combination antiretroviral therapy (cART), and are the major barrier to cure [1–3]. As these latently infected cells are scarce in patient blood [1, 2], *in vitro* models of HIV latency in resting CD4+ T-cells are essential to understand how latency is established, maintained and reversed, and develop new interventions.

Latency can be established *in vitro* by direct infection of resting CD4+ T-cells in the presence of stimuli, including the chemokine CCL19 [4–6]; high viral titres with or without spinoculation [7–11]; or culturing T-cells in contact with myeloid dendritic cells [mDC, [12]] or endothelial cells [13]. Some studies [4–6, 14], but not all [7], report that pre-conditioning resting CD4+ T-cells with the chemokine CCL19 enhanced direct infection of resting CD4+ T-cells *in vitro*. One study that reported that CCL19 was not required to establish latent infection *in vitro*, typically used high titres of infectious HIV up to a multiplicity of infection (MOI) of 3, with or without spinoculation [7]. Spinoculation has been reported to increase virus binding and alter the cell cytoskeleton, as well as enhance the efficiency of infection [15–17].

We have previously shown that chemokines that bind to chemokine receptors highly expressed on resting CD4+ T-cells can facilitate the establishment of latent infection *in vitro* via enhanced efficiency of nuclear localisation and integration [4]. HIV similarly binds the chemokine receptor CCR5 (R5) or CXCR4 (X4) as a co-receptor for entry [18–20]. As both events induce chemokine receptor signalling and changes in the actin cytoskeleton [21–25], we hypothesised that infecting resting CD4+ T-cells with high viral titres might enhance chemokine receptor signalling and bypass the need for CCL19. Therefore, we tested the impact of viral titre, co-receptor usage and donor variation on establishing HIV latency in resting CD4+ T-cells cultured alone, or pre-stimulated with CCL19 or mDC to enhance latency *in vitro*. We show that the establishment of latency *in vitro* through direct infection of resting CD4+ T-cells is significantly enhanced by CCL19 and mDC, but the efficiency was dependent on virus titre, co-receptor usage and there was significant donor variability.

## Materials and Methods

### Ethics Statement

The use of blood packs from healthy human donors from the Australian Red Cross Blood Bank for this study was approved by the University of Melbourne Office for Research Ethics and Integrity (Ethics ID: 1443071).

### HIV Plasmids, Viral Stocks and TCID_50_ determination

HIV plasmids: pNL4.3, pNL4.3-EGFP or pNL4.3(AD8)-EGFP were provided by Damian Purcell and Yasuko Tsunetsugu-Yokota [26, 27] and prepared using Qiagen Maxi Prep kits. Viral stocks were prepared by FuGene 6 (Promega, USA) transfection using 16 μg plasmid per T75cm^2^ flask of 293T cells [28]. Virus-containing media was collected at 24–36hr post-transfection, filtered (0.22 μm), ultracentrifuged through 20% sucrose, viral pellets resuspended in a 60-fold smaller volume and single-use aliquots stored at -80°C. The 50% tissue culture infectious dose (TCID_50_) of virus stocks was determined by diluting virus stocks 10-fold in a 96 well plate in triplicate and adding 2x10^5^ activated PBMCs [10 μg/ml phytohemagglutinin (PHA) plus 10 U/ml interleukin-2 (IL-2), Roche] pooled from 2 donors per well. Culture media was analysed after 7 days for HIV reverse transcriptase (RT) activity. Virus dilutions were scored as positive or negative if they were > or ≤ 2-fold the average RT in the no virus controls respectively, and the scores were used to determine TCID_50_/ml [29].

### HIV Reverse transcriptase (RT) Assay

RT activity in HIV stocks and T-cell culture media was quantified using a radioactive assay for intra-virion RT enzyme modified to use MgCl_2_ for HIV RT in place of MnCl_2_ for Moloney murine leukemia virus RT [30]. Concentrated HIV stocks were tested in a 2-fold dilution series due to high viral titres and results that fell in the linear assay range were used to determine RT.

### Isolation of PBMCs, resting CD4+ T-cells and myeloid dendritic cells

PBMCs were isolated from the blood of healthy volunteers (Australian Red Cross Blood Bank) via Ficoll-Paque density centrifugation. Resting CD4+ T-cells and myeloid dendritic cells (mDC) were then isolated as published [5, 12, 31], with purities >95% and >98% respectively.

Resting CD4+ T-cells were negatively selected using a monoclonal antibody cocktail targeting: CD8 (OKT-8 hybridoma, ATCC); CD11b (OKM-1 hybridoma, ATCC); CD14 (FMC-17 hybridoma, ATCC); CD16 (3G8 hybridoma, ATCC); glycophorin-A (10FM.N hybridoma, ATCC); CD69 (BD Biosciences, #347820); CD19 (FMC-63 hybridoma, Heidi Zola, Flinders Medical Centre, Adelaide, AUS) and HLA-DR (2.06 hybridoma, Heidi Zola, Flinders Medical Centre, Adelaide, AUS). Goat anti-mouse IgG microbeads (Miltenyi Biotech, #130-048-401) were added and an autoMACS (Milenyi Biotec) then used for magnetic bead depletion.

mDC were isolated in two steps. First negative selection was used with a monoclonal antibody cocktail targeting CD3 (OKT-3 hybridoma, ATCC), CD11b (OKM-1 hybridoma, ATCC) and CD19 (FMC-63 hybridoma, Heidi Zola, Flinders Medical Centre, Adelaide, AUS), plus goat anti-mouse IgG microbeads and an autoMACS (Miltenyi Biotech). Next, the negative fraction was stained with HLA-DR.APC-Cy7 (BDBiosciences, #335796), CD11c.V450 (BD Biosciences, #560369) plus CD123.PE (BD Biosciences, #555644) and sorted for HLA-DR+CD11c+ mDC using a FACSAria (BD Biosciences).

Individual hybridomas were cultured in RF10 (RPMI-1640, 10% foetal bovine serum, 1 U/ml Penicillin, 1 μg/ml Streptomycin, 2.92 μg/ml L-Glutamine) and culture supernatants collected and titrated on healthy donor PBMCs to determine hybridoma volumes required for cell selection.

T-cell purity was assessed using CD4.FITC (BD Biosciences, #555346) and CD3.PE (BD Biosciences, #555340) staining followed by analysis on a FACSCalibur (BD Biosciences).

### CCL19 resting CD4+ T-cell Latency Model with wild-type HIV^NL4.3^

Resting CD4+ T-cells were cultured in RF10 either alone, with 30nM or 100nM CCL19 for 24 hours, or activated for 2 days with 10 μg/ml PHA and 10 U/ml IL-2. Recombinant human CCL19/MIP-3β (R&D Systems, USA) was reconstituted in 0.1% BSA to 100 μg/ml and used fresh or from single use aliquots at -80°C. 1x10^6^ cell aliquots were incubated with 100ul HIV^NL4.3^ dilution for 2 hours at 37°C, washed 3 times in Dulbecco’s phosphate buffered saline (PD) and cultured in RF10 plus 1 U/ml IL-2. At Day 4, RT in culture media was measured. Intracellular genomic DNA (Qiagen Blood and Cell Culture DNA Mini kit) was analysed via qPCR (Agilent Technologies MX3000P) for integrated HIV DNA (Alu-LTR) and CCR5 to normalise integration values for cell number (Agilent Technologies MX3000P qPCR: [32].

### Comparison of CCL19 and mDC co-culture resting CD4+ T-cell Latency Models using HIV^NL4.3-EGFP^ or HIV^NL4.3(AD8)-EGFP^

Resting CD4+ T-cells, labelled with 5 μM eFluor670 cytoplasmic dye (eBiosciences, USA), were cultured for 24 hours in RF10 plus 2 U/ml IL-2 alone, with 100 nM CCL19 or autologous mDC (1 mDC:10 T-cell ratio). Cells were incubated with HIV^NL4.3-EGFP^ or HIV^NL4.3(AD8)-EGFP^ for 2 hours at 37°C, washed and cultured for 5 days in RF10 plus 2 U/ml IL-2. At Day 5 post-infection, T-cells were analysed for EGFP expression (productive infection) and eFlour670^hi^EGFP- T-cells sorted (FACSAria, BD Biosciences). 1x10^5^ sorted cell aliquots were cultured for a further 3 days (Day 8) in 96-well plates in 200 μl RF10 plus 2 U/ml IL-2 either alone (spontaneous EGFP expression) or in wells coated with 5 μg/ml anti-CD3 plus 5 μg/ml anti-CD28 and 50 ng/ml IL-7 also added to the media, to reactivate latent infection. 1 μM L-870812 integrase inhibitor (L8, kind gift from Merck, New Jersey, USA, [33]) was included where indicated to block integration in the CCL19 model [34] and measure reactivation of post-integration latency [35]. Cells were analysed for EGFP expression by flow cytometry (FACSCalibur, BD Biosciences) at Day 8.

### Statistical Analyses

GraphPad PRISM version 6 software was used for statistical analyses, using Spearman correlation (TCID_50_/ml versus RT/ml), Mann-Whitney or Wilcoxon matched-pairs signed rank (comparing cell types). *P*-values below 0.05 were considered significant.

## Results

### The requirement for CCL19 to establish latent infection *in vitro* is donor dependent

To quantify the infectious titre of virus for latency experiments, we had previously normalised virus input using reverse transcriptase (RT) activity per ml of viral stock [4, 5, 14]. However, the RT activity of virus may not reflect the infectivity of the virus on primary cells. Therefore, in this study, the infectivity of the virus inoculum was also measured using activated peripheral blood mononuclear target cells (PBMCs) and a limiting dilution assay to determine the 50% tissue culture infectious dose (TCID_50_). We found no direct correlation between TCID_50_ and RT activity in the same viral inocula (S1 Fig). Limiting dilution infection of activated PBMCs was used to measure the infectivity of virus in all subsequent experiments.

To test the impact of viral inoculum on resting CD4+ T-cells in the presence or absence of CCL19, resting CD4+ T-cells from healthy individuals were either cultured in media alone (unstimulated), pre-treated for 24 hours with 100 nM CCL19, or activated for 2 days with phytohemagglutinin (PHA) and interleukin-2 (IL-2). Cells were incubated with increasing viral titres of X4-tropic HIV^NL43^ for 2 hours, washed and cultured in media containing a low concentration of IL-2 (1 U/ml). These cells were analysed 4 days later for HIV integration, using Alu-LTR quantitative real-time PCR (qPCR), and virion production, by quantification of RT in the culture supernatant. Measurement of HIV integrated DNA in cells demonstrates the frequency of infected cells and an increase in viral RT in culture supernatant indicates productive viral replication in infected cells. We define HIV latency as HIV integration with no increase in RT in culture supernatant.

**Fig 1:**
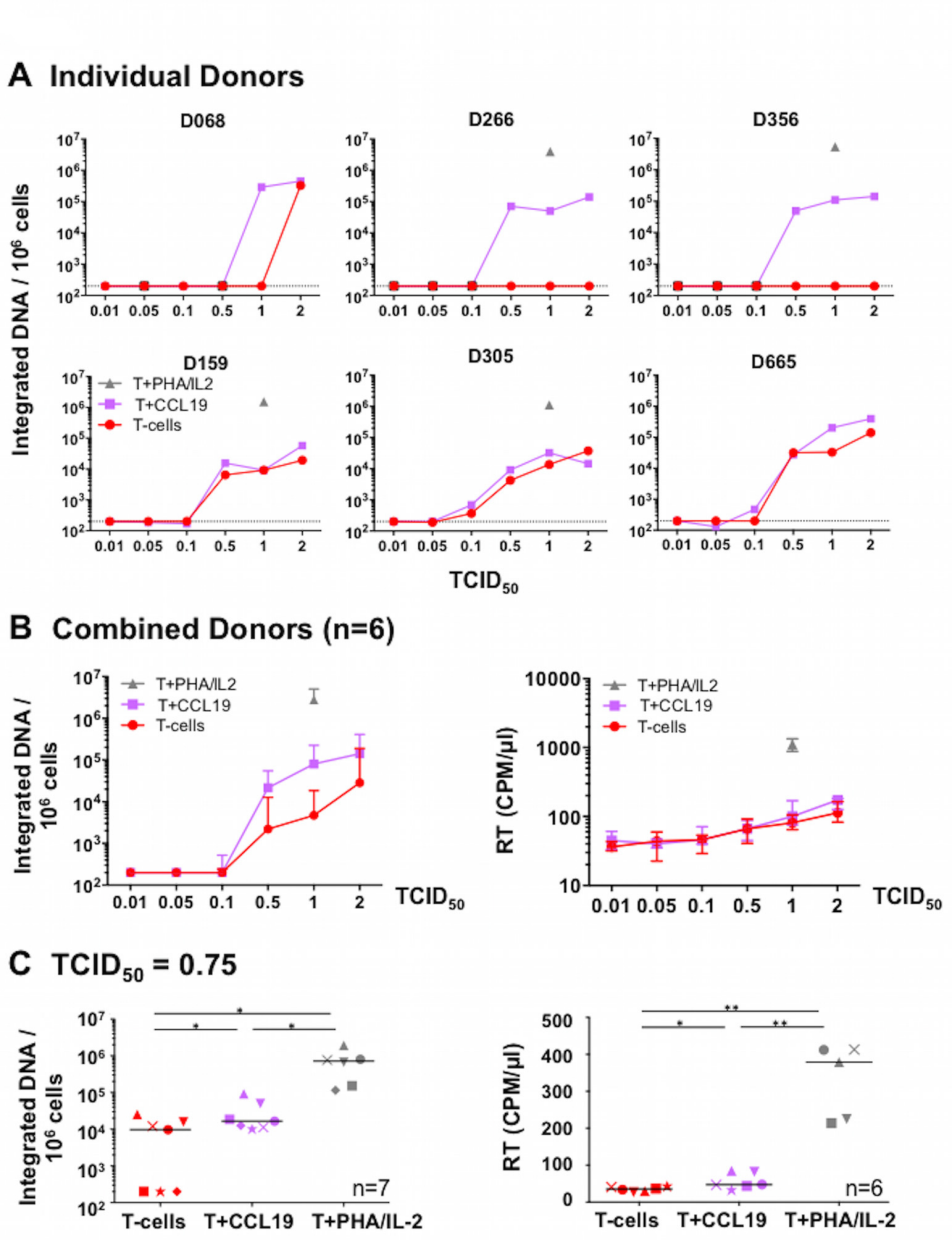
Viral titre and donor variability impacts HIV^NL4.3^ infection of unstimulated and CCL19-treated resting CD4+ T-cells. (A-B) Resting CD4+ T-cells from different donors were cultured unstimulated, pre-treated for 24 hours with 100 nM CCL19 or activated for 2 days with PHA+IL-2. Cells were incubated with different HIV^NL4.3^ doses for 2 hours, washed and cultured 4 days in media containing a low concentration of IL-2 (1 U/ml). Quantification of integrated HIV DNA by Alu-LTR qPCR is shown in A, individual donors and B *left panel*, the donors combined. Culture media was also analysed for released virus using a radioactive, HIV reverse transcriptase (RT) assay [counts per minute per μl of virus-containing media (CPM/μl) shown]. Median plus interquartile range for combined donor results is shown in B, *right panel*). (C) A similar experiment was performed using resting CD4+ T-cells from 7 different donors, except 30 nM CCL19 was used and cells were incubated with 0.75 TCID_50_ per cell of HIV^NL4.3^. At Day 4, cells were analysed for integrated HIV DNA by Alu-LTR qPCR *(left panel)* and RT quantified in cell culture media [(CPM/μl) shown]. Individual donors are shown as different symbols and the horizontal bar represents the median. Statistically significant differences in HIV integration or RT activity between cell types are shown as: *P < 0.05, **P < 0.01 (Wilcoxon matched-pairs signed rank test).

Higher titres of HIV^NL4.3^ led to an increase in the frequency of HIV integration (Fig 1A). Integration occurred in resting CD4+ T-cells without CCL19 in 3 of 6 donors at a TCID_50_ of 0.5–2 (Fig 1A: D159, D305 and D665). Conversely, in the other 3 donors, the presence of CCL19 enhanced the frequency of HIV integration compared to unstimulated cells. When we compared the data from all donors, the frequency of integration was increased in CCL19-treated versus unstimulated cells using virus stocks with a TCID_50_ of 0.5–2 (Fig 1B *left*), and the difference was greatest and statistically significant with a TCID_50_ of 1 (Mann-Whitney test, p = 0.015). Resting CD4+ T-cells, with or without CCL19, were not susceptible to infection using virus stocks with a TCID_50_ <0.5, as measured by lack of integration and low RT (Fig 1A and 1B).

For all 6 donors, increasing TCID_50_ led to a small, dose-dependent increase in RT activity in the culture supernatant of both unstimulated and CCL19-treated cells (Fig 1B *right*). However, this RT increase was modest compared to the high RT production observed following infection of T-cells activated with PHA/IL-2.

As our previous work showed that CCL19 could induce HIV latency at concentrations as low as 10 nM [5], we also examined the ability of HIV^NL4.3^ to infect a further 7 donors using a lower, 30nM concentration of CCL19 and an HIV^NL4.3^ TCID_50_ of 0.75 per cell. In 3 out of these further 7 donors, integration was only observed in CCL19 treated, and not in the unstimulated, resting CD4+ T-cells (Fig 1C *left panel*, T-cells versus T-cells+CCL19). RT remained low in the supernatants from all CCL19-treated cells in the presence of integration, consistent with latent infection (Fig 1C *right panel*).

Together, these data revealed that direct infection of resting CD4+ T-cells, in the absence of additional stimuli, is possible in some donors at higher viral titres. However, in those donors where direct infection was not established in the unstimulated cells (*eg*. Fig 1A: D266, D356), the addition of CCL19 facilitated HIV integration and the establishment of latency.

### Comparison of CCL19 and myeloid dendritic cells in establishing infection of resting CD4+ T-cells *in vitro*

**Fig 2:**
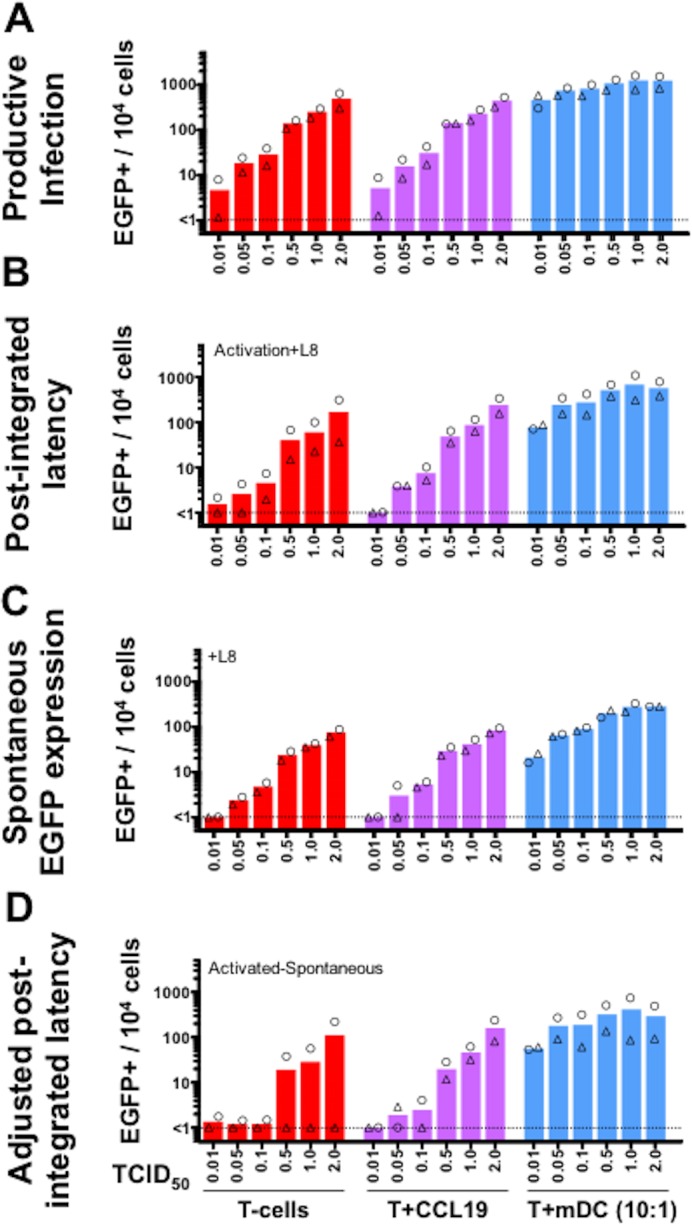
Latency is established with higher efficiency following co-culture of resting CD4+ T-cells with mDC. Resting CD4+ T-cells from 2 donors were labelled with eFluor670 cytoplasmic dye and cultured for 24 hours either untreated (*red*), with 100 nM CCL19 (*purple*) or with autologous mDCs at 1 mDC:10 T-cell ratio (*blue*). Cells were incubated with increasing TCID_50_ per cell of X4-tropic HIV^NL4.3-EGFP^. After 2 hours, cells were washed, cultured for 5 days in a low concentration of IL-2 (2 U/ml) and analysed for EGFP expression by flow cytometry to quantify productive infection (A). Additionally, non-proliferating eFluor670^hi^EGFP- cells were sorted, cultured for 3 days with anti-CD3/anti-CD28 plus IL-7, in the presence of the integrase inhibitor L-870812 (L8), to induce EGFP expression from post-integration latent infection (B). As a comparative control, aliquots of sorted cells were also cultured for 3 days with L-870812 (L8) but no reactivation stimuli in order to measure background, spontaneous EGFP expression during 3 further days of culture (C). The true level of post-integrated latency in cultures was calculated by subtracting the percentage of EGFP+ cells in the spontaneous cultures from the percentage of EGFP+ cells in reactivated cultures (D). The percentage of EGFP+ cells per 10^4^ cultured cells is shown. Columns represent the median of donor pairs with each donor shown as a different symbol.

We next compared the impact of virus inoculum on establishing latent infection of resting CD4+ T-cells in the presence of CCL19 versus another exogenous signal, co-culture with mDC [12]. Resting CD4+ T-cells were cultured alone, treated with CCL19 or co-cultured with autologous mDC for 24 hours. Cells were then incubated with HIV^NL4.3-EGFP^ using viral inoculums with a TCID_50_ ranging from 0.01 to 2. HIV^NL4.3-EGFP^ contains an enhanced green fluorescent protein (EGFP) reporter downstream of the viral *envelope* gene, and therefore EGFP expression can be used as a marker for HIV protein expression and productive infection [26]. After 2 hours, cells were washed and cultured 5 days in a low concentration of IL-2 (2 U/ml). At Day 5 post-infection, we saw a dose-dependent increase in productive infection in all three culture conditions (Fig 2A).

To measure inducible latent virus in cultures, at Day 5 post-infection, non-proliferating EGFP- cells were sorted to remove the productively infected (EGFP+) cells. The EGFP- cells were then activated with anti-CD3/anti-CD28/IL-7 to reactivate latent infection. EGFP was quantified after 3 days as a measure of inducible latent infection (Fig 2B). In these experiments, the integrase inhibitor L-870812 (L8) was included so that only EGFP expression from integrated provirus (post-integration latency) was quantified. EGFP expression in the absence of an activating stimulus was also quantified in the sorted cells to account for spontaneous (non-induced) EGFP expression (Fig 2C). Spontaneous EGFP expression was subtracted from EGFP expression observed following anti-CD3/anti-CD28/IL-7 activation (Fig 2B) to calculate adjusted post-integration latency (Fig 2D).

We observed a dose-dependent increase in post-integration latency, spontaneous EGFP expression and adjusted post-integration latency in unstimulated, CCL19-treated T-cells and in T-cells co-cultured with mDC (Fig 2B–2D). Co-culture with mDC induced a higher frequency of latent infection in CD4+ T-cells compared to both unstimulated and CCL19-treated T-cells. Similar to results using wild type X4-tropic HIV^NL4.3^, adjusted post-integration latency was not detected in unstimulated cells without CCL19, in 1 of 2 donors at a TCID_50_ of 0.5–2 (Fig 2D, triangle donor).

### High titres of R5-tropic HIV are needed to infect resting CD4+ T-cells *in vitro*

**Fig 3:**
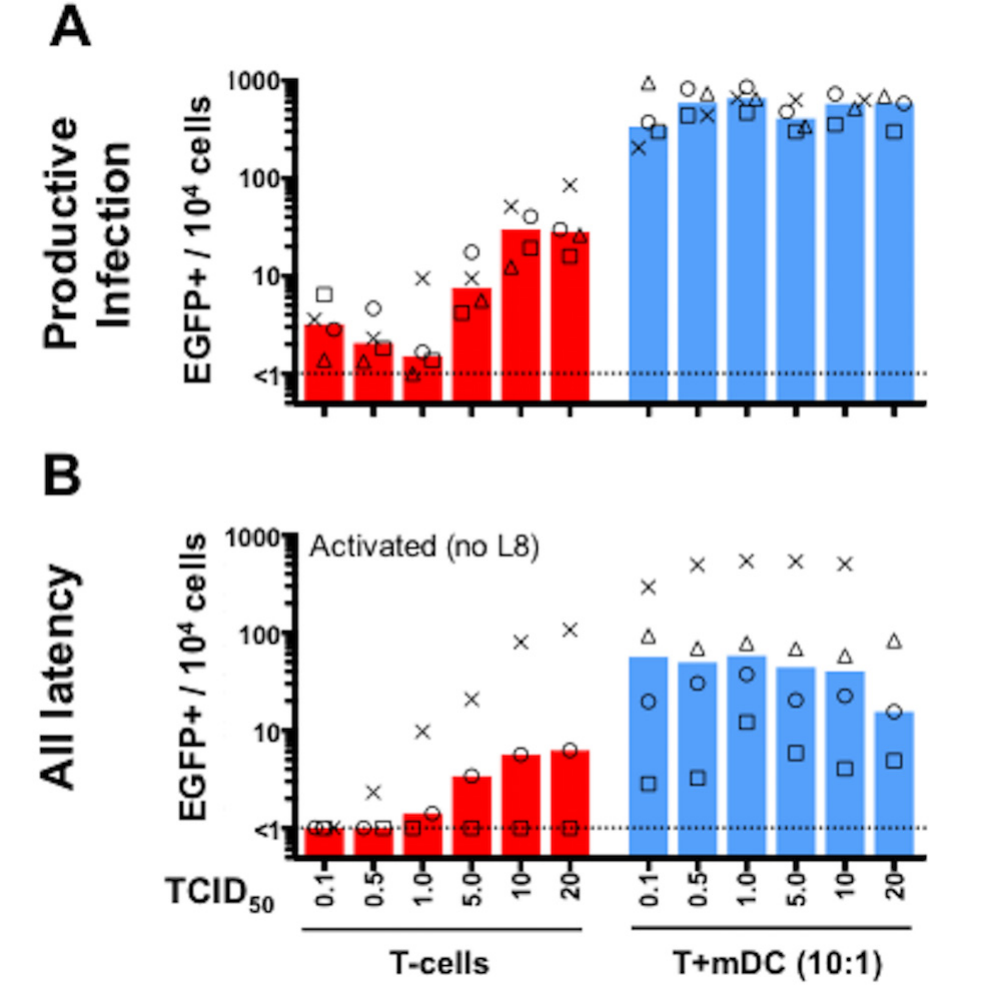
High titres of R5-tropic HIV.EGFP reporter virus are needed to infect resting CD4+ T-cells. Resting CD4+ T-cells from 4 donors were labelled with eFluor670 proliferation dye and cultured without (*red*) or with autologous mDCs at 1 mDC:10 T-cell ratio (*blue*) for 24 hours. Cells were incubated with increasing TCID_50_ per cell of HIV^NL4.3(AD8)-EGFP^ for 2 hours and cultured for 5 days in a low concentration of IL-2 (2 U/ml). 5 days post-infection, cultures were analysed for EGFP expression by flow cytometry to quantify productive infection (A). Non-proliferating eFluor670^hi^EGFP- cells were also sorted and cultured for 3 days with anti-CD3/anti-CD28 plus IL-7 to induce EGFP expression from latent infection (B). The percentage of EGFP+ cells per 10^4^ cultured cells is shown. Columns represent the median of donors tested with each donor shown as a different symbol.

Given that R5-tropic HIV are the major transmitted viral strain [36–38], and therefore are the main strains that establish HIV latency [10], we also assessed the impact of increasing R5-tropic HIV^NL4.3(AD8).EGFP^ titre on latent infection of unstimulated and mDC co-cultured resting CD4+ T-cells (Fig 3). mDCs potently induced productive (Fig 3A) and latent infection (Fig 3B) of resting CD4+ T-cells at all titres of R5-tropic HIV^NL4.3(AD8).EGFP^, demonstrating the high efficiency of this system to facilitate infection of resting CD4+ T-cells. In contrast, in resting CD4+ T-cells alone, R5-tropic HIV^NL4.3(AD8).EGFP^ with a TCID_50_ of ≥5 typically established productive (Fig 3A) and latent infection (Fig 3B) in most donors. This was a higher TCID_50_ than what was required to establish latency using the X4-tropic HIV^NL4.3-EGFP^, where latency was established with a TCID_50_ of 0.5–2 in most donors.

## Discussion

To better optimise the methods to establish HIV latency in resting CD4+ T-cells *in vitro*, we examined the impact of HIV titre, co-receptor usage and donor variation on infection of resting CD4+ T-cells cultured using two previously described *in vitro* models of HIV latency. We demonstrated that while X4-tropic HIV can infect unstimulated resting CD4+ T-cells, up to half of the donors required the presence of CCL19 to establish latent infection. Compared to CCL19, autologous mDC were more potent at establishing latency in resting CD4+ T-cells, using both X4 and R5-tropic virus. A higher titre of R5-tropic, compared to X4-tropic, HIV was required to infect unstimulated resting CD4+ T-cells in most donors. The establishment of latency *in vitro* through direct infection of resting CD4+ T-cells is significantly enhanced by CCL19 and mDC, but the efficiency was dependent on virus titre, co-receptor usage and there was significant donor variability.

It is unclear why resting CD4+ T-cells from some, but not all, donors are permissive to direct infection without CCL19. Some potential explanations for this observation include donor differences in the expression of CXCR4 or CCR5, and the varying frequency of free HIV Envelope gp120 protein in the virus inocula. HIV or free gp120 can both enhance signalling of downstream pathways following ligation to either CXCR4 or CCR5 leading to modification of the actin cytoskeleton [21, 23, 24], which is important for entry of HIV into the nucleus of a resting T-cell [4, 21]. Increased expression of donor CXCR4 or CCR5, and/or free gp120 in virus inocula could enhance permissiveness of resting T-cells to HIV infection, in the absence of CCL19. Alternatively, it is possible that during culture of T-cells from some donors, endogenous production of chemokines such as CXCL9 or CXCL10 (ligands for CXCR3) [12], or CXCL12 or extracellular ubiquitin from damaged cells (both ligands for CXCR4) [39, 40] may be expressed. These ligands could then potentially activate the relevant chemokine receptors and signalling pathways, similar to the effect of CCL19, to enable infection as we and others have previously shown [4, 21]. Finally, donor differences in the expression or phosphorylation/activity of cellular SAMHD1, which restricts HIV infection in resting CD4+ T-cells [41–43], may also impact the relative sensitivity or resistance of resting CD4+ T-cells to infection and the subsequent establishment of latency, and therefore the need for an additional stimulus such as exogenous chemokine such as CCL19. In these experiments, three experienced scientists performed these experiments using multiple different donors and each found similar results–that roughly half of the donors required the presence of CCL19 to facilitate infection of resting CD4+ T-cells.

While several other studies found that resting CD4+ T-cells were refractory to HIV infection *in vitro* [44, 45], our data is consistent with other studies that showed direct infection of resting CD4+ T-cells, and the establishment of latency *in vitro*, was possible but is inefficient and highly variable [6–11]. One group described that infection of resting CD4+ T-cells was possible without CCL19 in all donors [7]. The differences between our work and Pace et al. may lie in different cell isolation methods, or different methods used to determine viral titre and ultimately different viral titres used between studies. The high viral titre of MOI = 3 used in studies by Pace et al. might have bypassed the requirement for CCL19 in all donors. The use of spinoculation at the lower viral titre of MOI = 0.2 by Pace et al, which was not used in our studies, might also have enabled infection without CCL19 and could explain the different requirements for CCL19 between studies.

Despite previous studies showing that unstimulated and CCL19-treated resting CD4+ T-cells lack activation markers [5, 12] that are required for productive infection, one important issue we aimed to address in these experiments was whether there was low level productive infection in this model. In this study, productive infection was measured by quantifying RT in supernatant (Fig 1) or EGFP+ cells as a surrogate marker for HIV protein expression (Figs 2 and 3). Using X4-tropic HIV with a TCID_50_ of 0.5–2, we observed minimal change in RT (Fig 1), but EGFP+ cells were observed in all culture conditions using similar viral inocula (Fig 2A). The discrepancy between these two assays may be related to the RT assay measuring virions in culture media and EGFP expression measuring cell-associated viral protein. Despite expression of HIV protein (EGFP), insufficient viral proteins may have been made to generate virions, and thus RT remained low in the culture media. Alternatively, EGFP may be a more sensitive measure of productive infection than the RT assay. Finally, it is possible that we observed EGFP expression from unintegrated viral DNA, causing EGFP expression despite low RT [46–50]. However, this option seems unlikely as viral gene expression from unintegrated HIV DNA is limited to the expression of early viral genes (*eg*. *nef* [48]) but EGFP in the HIV.EGFP reporter viruses used here was expressed downstream of the late HIV *envelope* gene [26].

mDCs were most potent at inducing latent and productive infection of resting CD4+ T-cells following infection with both X4 and R5-tropic HIV EGFP reporter viruses, compared to unstimulated or CCL19-treated T-cells (Figs 2 and 3). Binding of HIV gp120 to CXCR4 and CCL19 to its receptor CCR7 activate intracellular signalling that alters actin dynamics, facilitating nuclear localisation and infection of resting T-cells [4, 21]. DCs can also form a stable synapse with T-cells, involving signalling and actin rearrangements [51, 52]. It is possible that mDC induce more effective signalling in resting T-cells and/or actin rearrangement than HIV alone or CCL19, allowing for more efficient T-cell infection. In lymphoid tissues *in vivo*, mDC and resting CD4+ T-cells migrate in the presence of chemokines, such as CCL19 and CCL21, and interact [reviewed in [53]]. Together this may provide a favourable environment for ongoing infection of resting CD4+ T-cells, even in the presence of very low levels of HIV that could persist in HIV infected subjects on ART. This might, potentially occur in sanctuary sites in HIV-infected subjects, such as B-cell follicles in lymph node tissue [54].

Of note, cells in this study were cultured in media containing a low dose of IL-2 (1 U/ml in Fig 1 or 2 U/ml IL-2 in Figs 2 and 3) post-infection to enhance cell survival over the culture period, as we have previously reported [4, 5, 12, 55]. Whether the presence of low dose IL-2 enhanced the level of integration and infection observed in resting CD4+ T-cells (Fig 1 or Figs 2 and 3) is unclear. We believe this is unlikely to have contributed because these cells were not proliferating or activated, as we have previously reported [12].

To quantify the level of infectious virus in stocks for this study, intra-virion RT activity versus viral infectivity on activated PBMCs (TCID_50_) was initially compared. The lack of correlation between RT activity and TCID_50_ measurements for the same virus stocks across multiple investigators (S1 Fig) might be related to the fact that RT activity does not reflect the infectivity of virus stocks on target cells. Other factors can determine infectivity, including the level of functional HIV envelope protein (Env) on virions, which is not measured by the RT assay. Shedding of surface gp120^Env^ from underlying gp41^Env^ on virions [56], potentially via vigorous pipetting/handling of virus stocks, and/or leaving viral stocks in culture for a longer period of time before harvesting (allowing more time for gp120^Env^ to be shed [56]), might reduce the infectivity of virus stocks on PBMCs irrespective of RT activity.

In conclusion, CCL19 enhances latent infection of resting CD4+ T-cells in a subset of donors at viral TCID_50_ titres of 0.5–2. While not all donors required CCL19 to facilitate direct infection of resting CD4+ T-cells, adding CCL19 ensured consistent infection of resting CD4+ T-cells *in vitro*. In comparison, latency was enhanced in resting CD4+ T cells following co-culture with mDC in all donors and at all viral titres tested and with higher efficiency. Therefore several exogenous signals, including CCL19 and mDC, can facilitate the efficient, direct infection of resting CD4+ T-cells and establishment of latency. Furthermore, differences in establishing infection *in vitro* are seen with differences in viral titre, co-receptor usage and some donor variability. Optimising *in vitro* models of HIV latency will be important in the development of new strategies to eliminate the establishment of or reverse latency.

## Supporting Information

S1 Fig Relationship between RT and TCID_50_ in concentrated HIV inocula. The amount of virus in concentrated preparations of HIV^NL4.3^ (*black*), HIV^NL4.3-EGFP^ (*green*) and HIV^NL4.3(AD8)-EGFP^ (*blue*) was measured using a radioactive assay for virion reverse transcriptase (RT) and a 50% tissue culture infectious dose assay (TCID_50_) with activated PBMC target cells from 2 donors. RT activity/ml did not correlate with TCID_50_/ml across all virus stocks (Spearman r). Furthermore, no correlation was observed between RT activity/ml and TCID_50_/ml when the data was separated into the 3 different virus types: HIV^NL4.3^ (*P* = 0.210), HIV^NL4.3-EGFP^ (*P* = 0.242) and HIV^NL4.3(AD8)-EGFP^ (*P* = 0.450, Spearman r).
(TIF)
