# Supplementary figures and images for: Understanding Factors That Modulate the Establishment of HIV Latency in Resting CD4+ T-Cells In Vitro
Source: PLoS One. 2016 Jul 6;11(7):e0158778. doi: 10.1371/journal.pone.0158778 (PMC4934909; doi:10.1371/journal.pone.0158778)

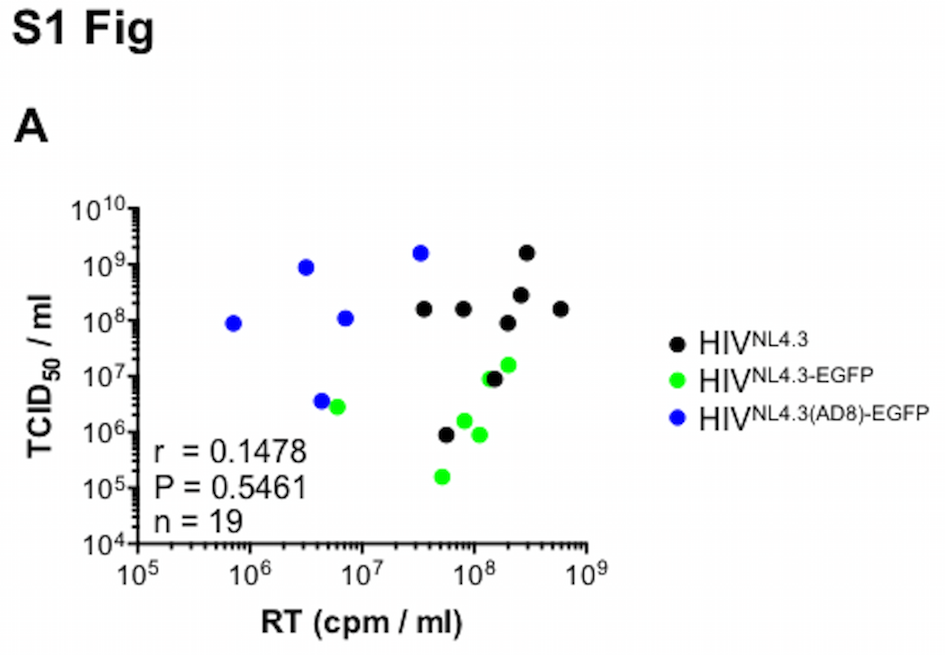

Supplement: S1 Fig — The amount of virus in concentrated preparations of HIVNL4.3 (black), HIVNL4.3-EGFP (green) and HIVNL4.3(AD8)-EGFP (blue) was measured using a radioactive assay for virion reverse transcriptase (RT) and a 50% tissue culture infectious dose assay (TCID50) with activated PBMC target cells from 2 donors. RT activity/ml did not correlate with TCID50/ml across all virus stocks (Spearman r). Furthermore, no correlation was observed between RT activity/ml and TCID50/ml when the data was separated into the 3 different virus types: HIVNL4.3 (P = 0.210), HIVNL4.3-EGFP (P = 0.242) and HIVNL4.3(AD8)-EGFP (P = 0.450, Spearman r). (TIF) [file pone.0158778.s001.tif]
